# PredCRG: A computational method for recognition of plant circadian genes by employing support vector machine with Laplace kernel

**DOI:** 10.1186/s13007-021-00744-3

**Published:** 2021-04-26

**Authors:** Prabina Kumar Meher, Ansuman Mohapatra, Subhrajit Satpathy, Anuj Sharma, Isha Saini, Sukanta Kumar Pradhan, Anil Rai

**Affiliations:** 1 ICAR-Indian Agricultural Statistics Research Institute, New Delhi, India; 2 Orissa University of Agriculture and Technology, Bhubaneswar, Odisha India; 3 Uttarakhand Council for Biotechnology, Pantnagar, Uttarakhand India

**Keywords:** Circadian clock, Circadian rhythms, Circadian genes, Computational biology, Machine learning

## Abstract

**Background:**

Circadian rhythms regulate several physiological and developmental processes of plants. Hence, the identification of genes with the underlying circadian rhythmic features is pivotal. Though computational methods have been developed for the identification of circadian genes, all these methods are based on gene expression datasets. In other words, we failed to search any sequence-based model, and that motivated us to deploy the present computational method to identify the proteins encoded by the circadian genes.

**Results:**

Support vector machine (SVM) with seven kernels, i.e., linear, polynomial, radial, sigmoid, hyperbolic, Bessel and Laplace was utilized for prediction by employing compositional, transitional and physico-chemical features. Higher accuracy of 62.48% was achieved with the Laplace kernel, following the fivefold cross- validation approach. The developed model further secured 62.96% accuracy with an independent dataset. The SVM also outperformed other state-of-art machine learning algorithms, i.e., Random Forest, Bagging, AdaBoost, XGBoost and LASSO. We also performed proteome-wide identification of circadian proteins in two cereal crops namely, *Oryza sativa* and *Sorghum bicolor*, followed by the functional annotation of the predicted circadian proteins with Gene Ontology (GO) terms.

**Conclusions:**

To the best of our knowledge, this is the first computational method to identify the circadian genes with the sequence data. Based on the proposed method, we have developed an R-package PredCRG (https://cran.r-project.org/web/packages/PredCRG/index.html) for the scientific community for proteome-wide identification of circadian genes. The present study supplements the existing computational methods as well as wet-lab experiments for the recognition of circadian genes.

**Supplementary Information:**

The online version contains supplementary material available at 10.1186/s13007-021-00744-3.

## Background

Rhythms of biological activity with a periodicity of 24 h are called circadian rhythms (CR) and are generated endogenously [1, 2]. There are molecular components with the underlying rhythmic features defining the circadian clock (CC). The three components (input, output and oscillator) model of the CC is the widely adopted one [3]. In this model, the input connects the environmental cues to the core component oscillator and the output links the functions of the oscillator with different biological processes [4]. So far, the CR has been extensively investigated in *Arabidopsis thaliana*, and the same clock mechanism has been extended to several dicot [5–8] and monocot [9, 10] plants as well.

The roles of CR in respect of regulating different metabolic pathways including carbon fixation and allocation of starch & sugar in leaf tissues have been reported in earlier studies [11, 12]. Anticipation of plants to environmental fluctuations (on a daily basis) is facilitated by CC [13], where the daily timing of the biological process is organized to specific time of the day and night [11, 14, 15] to increase the performance and reproductive fitness [16–18]. Including contribution to the agronomic traits of crops [19, 20], correct circadian regulations have been reported to enhance biomass accumulation, seed viability and photosynthesis [21, 22]. The roles of the circadian system in regulating plant response to different biotic and abiotic stresses have also been well studied [23, 24]. Plant growth and development related metabolisms are also regulated by CC, where it affects the quality and productivity of crops by bringing changes in the metabolites [25, 26]. The CC comprises several genes that form the transcriptional-translational feedback loops, resulting in rhythmic expression [11, 27]. The CC genes are reportedly involved in hormonal signaling [28, 29], growth and development of plant species [30, 31]. As reported in earlier studies [32, 33], crop productivity can be enhanced by manipulating the CC, particularly through circadian up-regulation of photosynthetic carbon assimilation.

A plethora of computational methods such as COSOPT [34], Fisher’s G-test [35], HAYSTACK [36], JTK-CYCLE [37], ARSER [38] and LSPR [39] have been developed for the identification of potential circadian genes using the gene expression data. A supervised learning approach ZeitZeiger [40] has also been developed for the identification of clock-associated genes from genome-wide gene expression data. In this study, we made an attempt to discriminate protein sequences associated with the circadian rhythms from the proteins that are not involved in the circadian clock. The motivations behind the present study are that (i) the existing computational methods use the genome-wide gene expression data for identifying the genes associated with the CC, (ii) identification of the circadian genes through wet-lab experiments require more time and resource, and (iii) no computational method based on the sequence (protein) data is available. In this study, we have employed the support vector machine with the Laplace kernel for discriminating circadian genes (CRGs) from non-CRGs by using the sequence dataset. We have also developed an R-package for easy prediction of CRGs by using the proteome-wide sequence data. This package is unique and we anticipate that our computational model will supplement the existing efforts for the identification of circadian genes in plants.

## Methods

### Collection of protein sequences

The protein sequences encoded by the experimentally validated oscillatory genes were collected from the Circadian Gene Database (CGDB) [41]. In this comprehensive database, about 73,000 genes encompassing 68 animals, 39 plants and 41 fungal species were available. A total of 12,041 protein sequences were retrieved from 9 plant species, i.e., *A. thaliana* (6981), *Glycine max* (4810), *O. sativa* (110), *Zea mays* (72), *Hodeum vulgare* (22), *Arabidopsis lyrata* (21), *Physcomitrella patens* (10), *Solanum tuberosum* (10) and *Triticum aestivum* (5). The 12,041 sequences were used to build the positive dataset. Further, 22,586 reviewed protein sequences of V*iridi plantae* collected from the UniProt (https://www.uniprot.org) were used to construct the negative dataset. The positive dataset thus comprised the protein sequences encoded by the circadian genes (CRG) and the negative dataset comprised the protein sequences encoded by other than the circadian genes (non-CRG). The positive and negative datasets were also referred to as CRG and non-CRG classes, respectively.

### Processing of positive and negative datasets

**Fig. 1:**
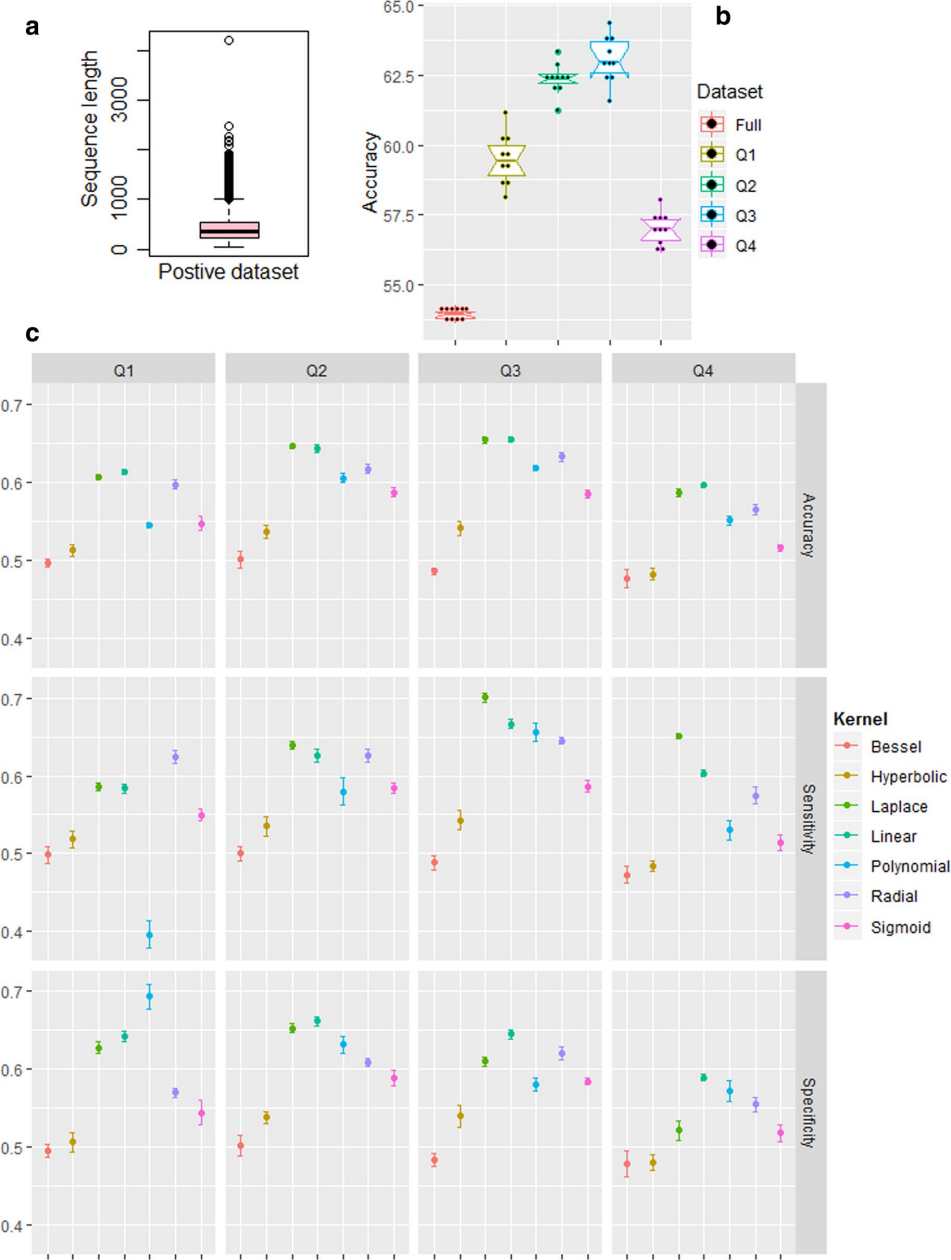
**a** Box plot of the sequence lengths of the positive dataset, where it can be seen that sequence length with more than 1000 amino acids are outlying observations. Thus, the maximum sequence length considered is 1000 amino acids. **b** Overall accuracy for the four homogeneous sub-datasets and the heterogeneous full dataset. It is seen that accuracies are higher for the sub-datasets with homogeneous sequence length as compared to dataset with highly heterogeneous sequence length. **c** Performance metrics for seven different kernel functions with respect to classification of circadian and non-circadian proteins using support vector machine. Among all the kernel functions, Laplace, linear and radial kernels are found to be superior with regard to overall classification accuracy

The CD-HIT program [42] was employed to remove the sequences that were > 40% identical to any other sequences. In order to avoid the homologous bias in the prediction accuracy, both positive and negative datasets were subjected to homology reduction. After removing the redundant sequences, 8211 and 6371 sequences were obtained for the negative and positive datasets, respectively. The sequences with residues B, J, O, U, X and Z were also excluded to avoid ambiguity for generating numeric features because these six letters do not stand for any of the amino acids that function as the building blocks of proteins. After removing such sequences, 8202 negative and 6370 positive sequences were retained for the analysis. It was also noticed that the lengths of the sequences in the positive dataset were highly heterogeneous (39–4218 residues). Thus, the positive dataset was divided into four homogeneous subsets (P1, P2, P3 and P4) based on quartile values of the sequence length in order to improve the prediction accuracy, where $39\le P1<221$, $221\le P2<363$, $363\le P3<538$ and $538\le P4<1001$(Table 1). Since the sequences with > 1000 amino acids were detected as outliers (Fig. 1a), using such sequences may generate noisy feature vectors. Hence, the sequences with > 1000 residues were further excluded from the analysis. Similar to the positive set, four subsets (N1, N2, N3 and N4) were created from the negative dataset, where $43\le N1<407$, $407\le N2<485$, $485\le N3<607$ and $607\le N4<1001$(Table 1). In this way, we prepared four homogeneous sub-datasets, i.e., Q1 (P1, N1), Q2 (P2, N2), Q3 (P3, N3) and Q4 (P4, N4) instead of a single heterogeneous dataset (Table 2).

**Table 1:** Summary statistics of the sequence length for positive and negative datasets

Dataset	Min	1^st^ Quartile	Median	3^rd^ quartile	Max
Positive	39	221	363	539	4218
Negative	43	256	407	607	5400

Based on the summary, both positive and negative datasets are divided into four sub datasets, where the length categories are minimum to 1st quartile, 1st quartile to median, median to 3rd quartile and 3rd quartile to 1000 (amino acids)

**Table 2:** Summary of the positive and negative datasets

Sub-dataset	#Positive sequence	#Negative sequence	Length category
Q1	1588	2045	*P1, N1*
Q2	1596	2047	*P2, N2*
Q3	1593	2050	*P3, N3*
Q4	1365	1499	*P4, N4*
Total (Full dataset)	6142	7641	-

Full dataset of positive and negative classes are partitioned into four sub-datasets i.e., Q1, Q2, Q3 and Q4. The partitioning was done based on the homogeneity of sequence length. For the Q1 sub-dataset, the sequence lengths for the positive and negative classes are P1 and N1 respectively, where P1 corresponds to 39 to 221 amino acids and N1 corresponds to 43 to 407 amino acids sequence length. Similar inference can be made for other sub-datasets

P1: 39 to 221 amino acids; P2: 221 to 363 amino acids; P3: 363 to 538 amino acids; P4: 538 to 1000 amino acids; N1: 43 to 407 amino acids; N2: 407 to 485 amino acids; N3: 485 to 607 amino acids; N4: 607 to 1000 amino acids

### Generation of numeric features

For each protein sequence, we generated amino acid composition (AAC), ProtFP features [43], FASGAI features [44], Cruciani properties [45], transitional properties [46, 47] and other physico-chemical properties (hydrophobicity, instability index, molecular weight and iso-electric point). The AAC is one of the popular features of protein sequences [48–51] which comprises a 20-dimensional numeric vector of amino acid frequencies. Given its simplicity and computational ease, the AAC is a well-performing feature set in terms of accuracy [51]. The ProtFP descriptor comprises the first 8 principal components obtained from the principal component analysis of 58 AAindex [52] properties of 20 amino acids. Based on the ProtFP features, each sequence was transformed into an 8-dimensional numeric feature vector. The FASGAI is a set of 6 numeric descriptors that represent 6 different properties of protein sequences, i.e., bulky properties, hydrophobicity, compositional characteristics, alpha and turn propensities, electronic properties and local flexibility. The Cruciani properties comprise 3 descriptors (polarity, hydrophobicity and H-bonding) that are based on the interaction of amino acids with different chemical groups. The transitional features represent the frequencies of amino acid residues of one type followed by residues of other types. Pertaining to transitional features, three types of residues for hydrophobicity (polar, neutral and hydrophobic), three types of residues corresponding to secondary structure (strand, helix and coil) and two types of residues for solvent accessibility (exposed and buried) were utilized. By using 8 types of residues, a total of 21 transitional descriptors were generated for each protein sequence. After combining all the feature sets, a total of 62 numeric features were obtained. A brief description about these features and the R-packages used to generate these features are provided in the Additional file 1: Table S1.

### Prediction with support vector machine

Support vector machines (SVM) [53] have been widely and successfully employed in the field of bioinformatics [54–60], and hence we have utilized the SVM for prediction in the present study. Binary SVM classifier was employed for the classification of CRG and non-CRG proteins. Let ${x}_{i}$ be the 62-dimensional numeric feature vector for the *i*^*th*^ protein sequence, where *i* = 1, 2, …, *N*. Further, *N*_*1*_ and *N*_*2*_ are the respective number of protein sequences for the CRG and non-CRG classes such that *N* = *N*_*1*_ + *N*_*2*_. Also, let us denote ${y}_{i}$ as the class label for ${x}_{i}$, where ${y}_{i}\in${-1, 1} with 1 and -1 as the class labels for the CRG and non-CRG classes, respectively. The decision function for the binary SVM classifier to classify a new observation vector $x$ can be formulated as.

$$f\left(x\right)=sgn\left\{\sum_{i=1}^{N}{y}_{i}{\alpha }_{i}K\left({x}_{i}, x\right)+b\right\}.$$

The value of ${\alpha }_{i}$ can be obtained by solving the convex quadratic programming

$$maximize \sum_{i=1}^{N}\sum_{j=1}^{N}{\alpha }_{i}{\alpha }_{j}{y}_{i}{y}_{j}K({x}_{i}{,x}_{j})$$

subjected to the constraint.

0≤$${\alpha }_{i}\le C$$ and $$\sum_{i=1}^{N}{\alpha }_{i}{y}_{i}=0$$.

Here, *C* is the regularization parameter that controls the tradeoffs between margin and misclassification error, and *b* is the bias term. Choosing an appropriate kernel function in SVM is important because the kernel function maps the input dataset to a high-dimensional feature space where the observations of different classes are linearly separable. In this study, 7 different kernel functions $K\left({x}_{i}{,x}_{j}\right)$ were utilized (Table 3). The performances of the kernels were first evaluated with the default parameters (Additional file 2: Table S2) by using a sample dataset. Then, the kernel functions with higher accuracies were chosen for the subsequent analysis.

**Table 3:** List of kernel functions and their mathematical expressions

Kernel type	Kernel function $\left\{{\varvec{K}}\left({{\varvec{x}}}_{{\varvec{i}}}{,{\varvec{x}}}_{{\varvec{j}}}\right)\right\}$
Radial basis function (RBF)	$exp(-\gamma {\| {x}_{i}-{x}_{j}\| }^{2})$
Polynomial	$(\gamma <{x}_{i}{,x}_{j}{>+r)}^{d}$
Linear	$<{x}_{i}{,x}_{j}>$
Hyperbolic	$tanh(\gamma <{x}_{i}{,x}_{j}>+r)$
Laplace	$exp(-\gamma \| {x}_{i}-{x}_{\mathrm{j}}\| )$
Bessel	$-{Bessel}_{order}^{d}\gamma {\| {x}_{i}-{x}_{\mathrm{j}}\| }^{2}$
Sigmoid	${(<{x}_{i}{,x}_{j}>+r)}^{d}$

$\gamma$, $d,$ $r$ and $order$ are kernel parameters and <  > denotes the inner product

### Cross-validation approach

In the present study, we employed fivefold cross-validation to control the bias-variance trade-off [61] and assess the performance of the SVM classification models. To perform the fivefold cross-validation, observations of CRG and non-CRG classes were randomly partitioned into 5 equal-sized subsets each. In each fold of the cross-validation, one randomly selected subset from each CRG and non-CRG classes were used as test set and the remaining four subsets of CRG and non-CRG classes together were used as training set. The classification was repeated five times with different training and test sets in each fold. The accuracy was computed by taking an average over all the five test sets.

### Prediction with balanced dataset

In all the four sub-datasets (Q1, Q2, Q3, Q4), the size of the negative set was higher than that of the positive set (Table 2). By using such an imbalanced dataset, the SVM classifier may produce biased accuracy towards the class having a larger number of instances. Thus, a balanced dataset was preferred for prediction using the SVM classifier. The balanced dataset was prepared by taking all the instances of the positive class and an equal number of instances from the negative class. For instance, the balanced dataset for Q1 contained 1588 positive and 1588 randomly drawn negative (from 2045) instances. Further, using only one random negative set means the remaining negative instances are out of the evaluation. To overcome such a problem, the classification experiment was repeated 10 times with a different negative set (randomly drawn) each time along with the same positive set. So, the problem of unbalanced-ness was handled by following the repeated cross-validation procedure, without training of the SVM model with unbalanced data. Performance metrics were measured by following the fivefold cross-validation technique and the final metrics were obtained by taking an average over all the 10 experiments.

### Using predicted class as a feature

The labels of each instance were represented as − 1 and 1 for the CRG and non-CRG classes respectively. The predicted labels of the instances obtained after classification was considered as a numeric feature and added to the existing feature set. Then, the prediction using the same dataset (with different training and test) was performed again by using the new feature set. This process was repeated 50 times and the accuracy was analyzed after adding the new feature each time. The idea of using the predicted label as numeric feature was implemented to achieve higher classification accuracy.

### Performance metrics

The true positive rate (TPR or *sensitivity*), true negative rate (TNR or *specificity*), *accuracy*, positive predictive value (PPV or *precision*), area under receiver operating characteristic curve (*auROC*) and area under precision-recall curve (*auRPC*) were computed to evaluate the performance of classifier. The *TPR*, *TNR*, *accuracy* and *PPV* are defined as follows.

$$Sensitivity \left(TPR\right)=\frac{TP}{TP+FN},$$

$$Specificity \left(TNR\right)=\frac{TN}{TN+FP},$$

$$Accuracy=\frac{1}{2}\left(TPR+TNR\right),$$

$$Precision \left(PPV\right)=\frac{TP}{TP+FP}.$$

The *TP* and *TN* are the number of correctly classified instances of the CRG and non-CRG classes, respectively. The FN and FP are the number of misclassified instances of the CRG and non-CRG classes, respectively. The *ROC* curve was obtained by taking the *sensitivity* in y-axis and *1-specificity* in x-axis, whereas the *PR* curve was plotted by taking the *precision* and *recall* (*sensitivity*) in x- and y-axes respectively.

## Results

### Prediction analysis with different sequence length category

Prediction was performed with the full dataset and sub-datasets, where 50% randomly drawn observations from both CRG and non-CRG classes were utilized. For comparing the accuracy between the full dataset (diverse sequence length) and sub-datasets (homogeneous sequence length), prediction was done only with the RBF kernel because the trend in accuracy between the homogeneous and full datasets was expected to remain the same by using the other kernels as well. The accuracies were observed to be higher (~ 4–6%) for the homogenous sub-datasets (Q1, Q2, Q3, Q4) as compared to the heterogeneous full dataset (Fig. 1b). Thus, the four sub-datasets (i.e., Q1, Q2, Q3 and Q4) were used hereafter instead of full dataset.

### Prediction analysis with different kernel functions

Performance of the kernel functions were compared by using a random sample of 50% observations. The sensitivity and specificity were respectively higher with the Laplace and linear kernels for the sub-datasets Q2, Q3 and Q4 (Fig. 1c). For sub-dataset Q1, sensitivity and specificity were higher with the RBF and polynomial kernels, respectively (Fig. 1c). The linear and Laplace kernels achieved similar accuracy for Q2 and Q3 sub-datasets, whereas the linear kernel achieved a little higher accuracy than the Laplace for Q1 and Q4 (Fig. 1c). Thus, no single kernel was found to perform better for each sub-dataset. It was also observed that the performance accuracies were higher for Q2 and Q3 (~ 65%) than that of Q1 and Q4 (~ 60%). Further, the Bessel kernel function achieved the lowest (~ 50%), followed by the hyperbolic kernel (Fig. 1c). As the Laplace, linear and RBF kernels achieved higher accuracies as compared to the other kernel functions, these three kernels were chosen for the subsequent prediction analysis. The mathematical representations of the Laplace and RBF functions are similar except for the distance between the feature vectors which is expressed in squared term for the RBF and in linear term for the Laplace. This may be the reason the variability captured by the Laplace kernel could be higher than that of RBF kernel, resulting in higher classification accuracy with the Laplace kernel. Further, the polynomial, hyperbolic and sigmoid kernels are the transformation of the linear kernel with additional parameters. So, the variability with respect to the discrimination of the CRG and non-CRG classes couldn’t be captured well by these kernels. This may be one of the possible reasons that the linear kernel achieved higher accuracy as compared to the other three kernels.

### Prediction analysis with iteratively added features

**Fig. 2:**
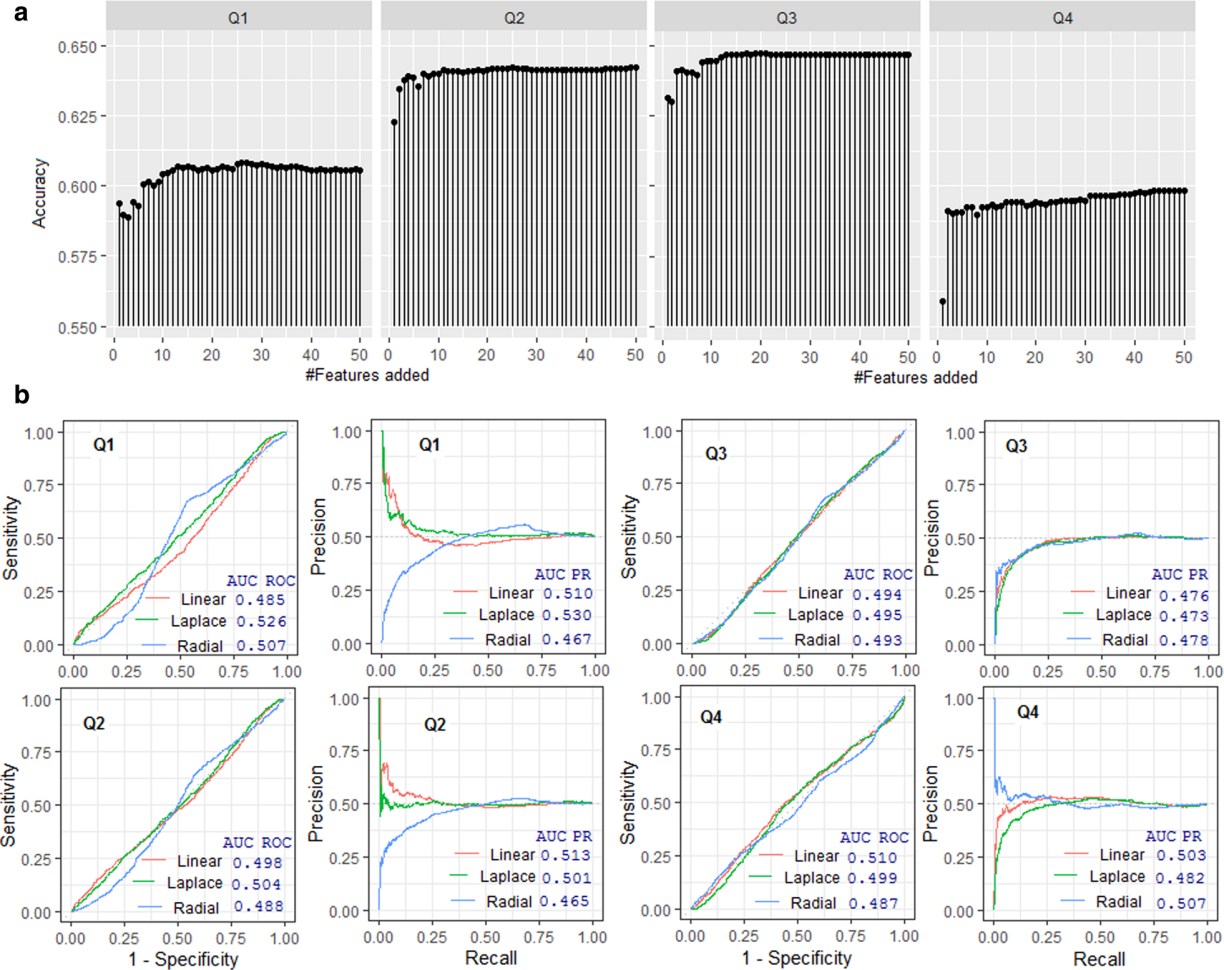
**a** Classification accuracy with respect to classification of circadian and non-circadian proteins by using support vector machine with the radial (RBF) kernel with addition of iteratively generated features. It is observed that the accuracies are improved by addition of iteratively generated features in all the four sub-datasets. **b** ROC and PR curves with regard to the classification of circadian and non-circadian proteins by using support vector machine with linear, Laplace and RBF kernels

Either a little or no improvement in accuracies were observed with the Laplace and linear kernels, even after adding 50 predicted label features (results not provided). On the other hand, 2–4% improvement in accuracies was observed with the RBF kernel after including the additional features. Specifically, accuracies in Q1, Q2, Q3 and Q4 reached plateau after addition of 26, 25, 20 and 45 features, respectively (Fig. 2a). The probable reason for not improvement in accuracy for the linear and Laplace kernels may be the variability introduced in the dataset with the inclusion of features (only -1 s and 1 s) was not captured well by these two kernels. On the other hand, the non-linear RFB kernel could have captured that variability which contributed towards the discrimination of both the classes. Nevertheless, accuracies of the linear and Laplace without iterated features and RBF with iterated features were found to be similar. Thus, we employed these three kernels for the subsequent prediction analysis.

### Final prediction analysis

Final prediction analysis was performed using the three selected kernels (Laplace, linear and RBF) with optimum parameters setting. The optimum values $\gamma$ (for RBF and Laplace) and *C* (for RBF, Laplace and linear) were determined by performing a grid search with $\gamma$: 2^–6^ to 2^6^ and *C*: 2^–6^ to 2^6^ with step size 2. Here, 2^–6^:2^6^ with step size 2 means 2^–6^, 2^–5^, 2^–4^, 2^–3^, 2^–2^, 2^–1^, 2^0^, 2^1^, 2^2^, 2^3^, 2^4^, 2^5^, 2^6^. For all the three kernels, higher accuracies were obtained with the default parametric values. Therefore, the prediction was made with the default parameter settings (Additional file 2: Table S2). Higher accuracies were obtained with the linear kernel for Q1 (61.13%) and Q2 (64.76%) sub-datasets, whereas the Laplace and RBF achieved higher accuracies for Q3 (65.69%) and Q4 (60.01%) respectively (Table 4). With regard to precision, the linear kernel achieved higher accuracies for Q1 (61.63%), Q2 (65.19%) and Q3 (64.67%), whereas the RBF kernel secured the highest accuracy for Q4 (60.05%) (Table 4). Sensitivities of Q1 (68.64%) and Q2 (67.52%) were higher with the RBF kernel, whereas the sensitivities for Q3 (70.56%) and Q4 (64.91%) were higher with the Laplace kernel (Table 4). Higher values of specificities were obtained with the linear kernel for Q2 (62.61%) and Q3 (63.89%), whereas the RBF and Laplace kernels achieved higher specificities for Q4 (60.29%) and Q1 (63.21%), respectively (Table 4). The aucROC values for Q1 (52.5%), Q2 (50.4%), Q3 (49.5%) were higher with the Laplace kernel, whereas the linear kernel secured higher aucROC for Q4 (51.1%) (Fig. 2b). The aucPR values for Q3 (47.8%) and Q4 (50.7%) were higher with the RBF kernel, whereas the Laplace and linear kernel achieved higher aucPR for Q1 (53%) and Q2 (51.3%), respectively (Fig. 2b).

**Table 4:** Classification accuracy of the support vector machine with three different kernels with default parameters

Dataset	Kernel	Sensitivity	Specificity	Accuracy	Precision
Q1	Linear	59.24 $\pm$ 0.90	63.02 $\pm$ 3.42	61.13 $\pm$ 1.66	61.63 $\pm$ 2.16
	Laplace	58.86 $\pm$ 1.71	63.21 $\pm$ 2.42	61.04 $\pm$ 1.86	61.56 $\pm$ 2.07
	Radial	68.64 $\pm$ 1.55	51.86 $\pm$ 3.42	60.25 $\pm$ 1.54	58.81 $\pm$ 1.55
Q2	Linear	63.32 $\pm$ 2.06	66.21 $\pm$ 1.39	64.76 $\pm$ 1.48	65.19 $\pm$ 1.43
	Laplace	64.07 $\pm$ 1.83	64.32 $\pm$ 2.11	64.20 $\pm$ 1.49	64.25 $\pm$ 1.57
	Radial	67.52 $\pm$ 3.67	60.62 $\pm$ 1.30	64.07 $\pm$ 1.93	63.14 $\pm$ 1.47
Q3	Linear	66.10 $\pm$ 4.34	63.89 $\pm$ 2.85	65.01 $\pm$ 2.20	64.67 $\pm$ 1.94
	Laplace	70.56 $\pm$ 4.25	60.81 $\pm$ 2.61	65.69 $\pm$ 1.93	64.29 $\pm$ 1.56
	Radial	67.61 $\pm$ 3.52	60.75 $\pm$ 4.74	64.18 $\pm$ 1.11	63.36 $\pm$ 1.78
Q4	Linear	59.26 $\pm$ 2.29	57.94 $\pm$ 3.71	58.61 $\pm$ 2.50	58.53 $\pm$ 2.63
	Laplace	64.91 $\pm$ 2.68	53.11 $\pm$ 2.27	59.01 $\pm$ 1.71	58.06 $\pm$ 1.48
	Radial	59.70 $\pm$ 2.52	60.29 $\pm$ 1.98	60.01 $\pm$ 1.51	60.05 $\pm$ 1.45

Classification was made with each sub dataset and performance metrics were computed following repeated cross validation where the experiment was repeated 100 times. In terms of accuracy, performances are higher for the Laplace kernel for Q2 and Q3 sub-datasets, whereas linear and RBF kernel performed better in Q1 and Q4 respectively. Performance metrics are higher for Q2 and Q3 sub-datasets than that of Q1 and Q4. The accuracies are seen to be more stable for RBF kernel, barring few exceptions

The linear kernel achieved higher accuracy and precision for Q1, whereas the aucPR, aucROC and specificity were higher with the Laplace kernel. For Q2, the specificity, accuracy, precision and aucPR were higher with the Laplace kernel, whereas the linear kernel achieved higher accuracy in terms of sensitivity and aucROC. In Q3, the specificity, precision and aucPR were higher with the linear kernel, whereas the sensitivity, accuracy and aucROC were higher with the Laplace kernel. For Q4, though RBF secured higher accuracy in terms of specificity, accuracy, precision and aucPR, the Laplace kernel achieved higher accuracy in terms of sensitivity and aucROC than that of RBF. Thus, no kernel was found to be an obvious choice with regard to higher prediction accuracy. Therefore, we employed a multiple criteria decision making (MCDM) approach to determine the best kernel function which is explained in the next section.

### TOPSIS analysis

The MCDM method TOPSIS [62] with different performance metrics as the multiple criteria was used to determine the best kernel (in terms of accuracy). The TOPSIS scores were higher with the Laplace kernel for Q1 (61.12) and Q3 (58.11), whereas the linear and RBF kernel achieved higher scores for Q2 (67.50) and Q4 (57.91) sub-datasets, respectively (Table 5). Overall, the highest score (73.20) was achieved by the Laplace kernel as compared to the linear (70.09) and RBF (23.77) kernel functions (Table 5). Thus, the Laplace kernel function was chosen as the best kernel function and utilized for the subsequent analysis.

**Table 5:** TOPSIS scores of the prediction performance for the three different kernels

Kernel	Q1	Q2	Q3	Q4	Overall
Linear	54.64	**67.50**	45.98	47.56	70.09
Laplace	**61.12**	59.85	**58.11**	41.67	**73.20**
Radial	40.78	31.75	24.98	**57.91**	23.77

For Q1 and Q3, TOPSIS scores are higher for the Laplace kernel, whereas linear and RBF achieved higher scores for Q2 and Q4 respectively. While all the four sub-datasets are accounted, the Laplace kernel achieved higher TOPSIS score than the other two kernel functions

### Prediction with the independent test dataset

The SVM with the Laplace kernel was used for the prediction of the independent dataset. The independent dataset was built with the circadian clock associated sequences collected from the existing studies. We collected 30 sequences from [63], 27 sequences from [64], 13 sequences from [33] and 26 sequences from [65]. Out of 96 sequences (30 + 27 + 13 + 26), some sequences were not found in NCBI (while searching with the gene ID) and some others were found to be present in the training (positive) dataset. After excluding such sequences, the remaining 54 circadian protein sequences were used as an independent dataset. Prediction for the independent dataset was made by using the models trained with Q1, Q2, Q3 and Q4 sub-datasets. Out of 54 sequences, 34 sequences were correctly predicted as circadian proteins and 20 sequences were wrongly predicted as non-circadian proteins. In other words, an accuracy of 62.96% was obtained with the independent dataset, which was similar to that of fivefold cross-validation accuracy with the Laplace kernel i.e., 62.48% (61.04 + 64.20 + 65.69 + 59.01/4). Thus, it may be said that the prediction accuracy was neither overestimated nor underestimated.

### Comparative analysis with other machine learning algorithms

**Fig. 3:**
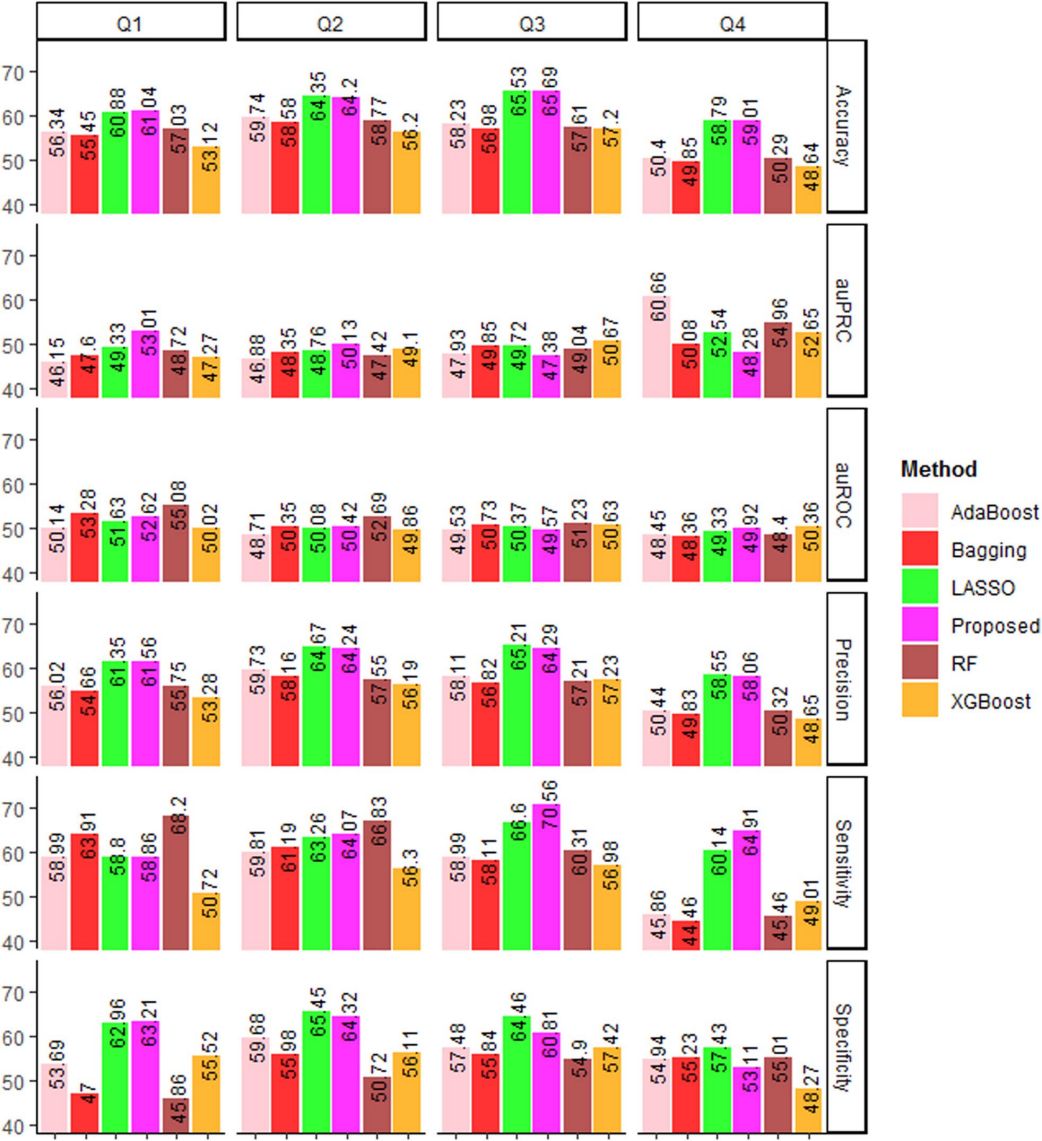
Difference performance metrics of the proposed approach (SVM with the considered features) along with the five other state-of-art learning algorithms. The accuracies of the proposed approach are found at par with that of LASSO, but higher than the other algorithms

The performance of SVM with the Laplace kernel (proposed approach) was further compared with that of other state-of-art machine learning algorithms, i.e., Random Forest (RF) [66], Bagging [67], Adaptive Boosting (AdaBoost) [68], eXtreme Gradient Boosting (XGBoost) [69] and L1-penalized logistic regression LASSO [70]. The RF, Bagging, AdaBoost, XGBoost and LASSO were implemented by using the R-packages *randomForest* [71], *ipred* [72], *adabag* [73], *xgboost* [74] and *glmnet* [75] respectively. All the predictions were made with default parameters (Additional file 3: Table S3) and the performance metrics were measured by following fivefold cross-validation. In terms of sensitivity, specificity, accuracy and precision, performance of the LASSO and the proposed approach were observed to be higher than that of other four algorithms (Fig. 3). RF achieved higher auROC for Q1 (55.08%), Q2 (52.69%) and Q3 (52.23%), whereas XGBoost for Q4 (50.36) sub-datasets (Fig. 3). The proposed approach achieved higher aucPRC for Q1 (53.01%) and Q2 (50.13%), whereas XGBoost and AdaBoost for Q3 (50.67%) and Q4 (60.66%), respectively. Between LASSO and the proposed approach, higher specificities were achieved by LASSO (Q2: 65.45%, Q3: 64.46%, Q4: 57.43%) than that of proposed approach (Q2: 64.32%, Q3: 60.81%, Q4: 53.11%). On the other hand, higher sensitivities were observed for the proposed approach (Q2: 64.07%, Q3: 70.56%, Q4: 64.91%) than that of LASSO (Q2: 63.26%, Q3: 66.6%, Q4: 60.14%). However, the accuracy and precision of the proposed approach and LASSO were found to be similar (Fig. 3). Thus, the LASSO and the proposed approach may achieve similar accuracy and better than the other considered algorithms.

### Proteome-wide identification and functional annotation

**Fig. 4:**
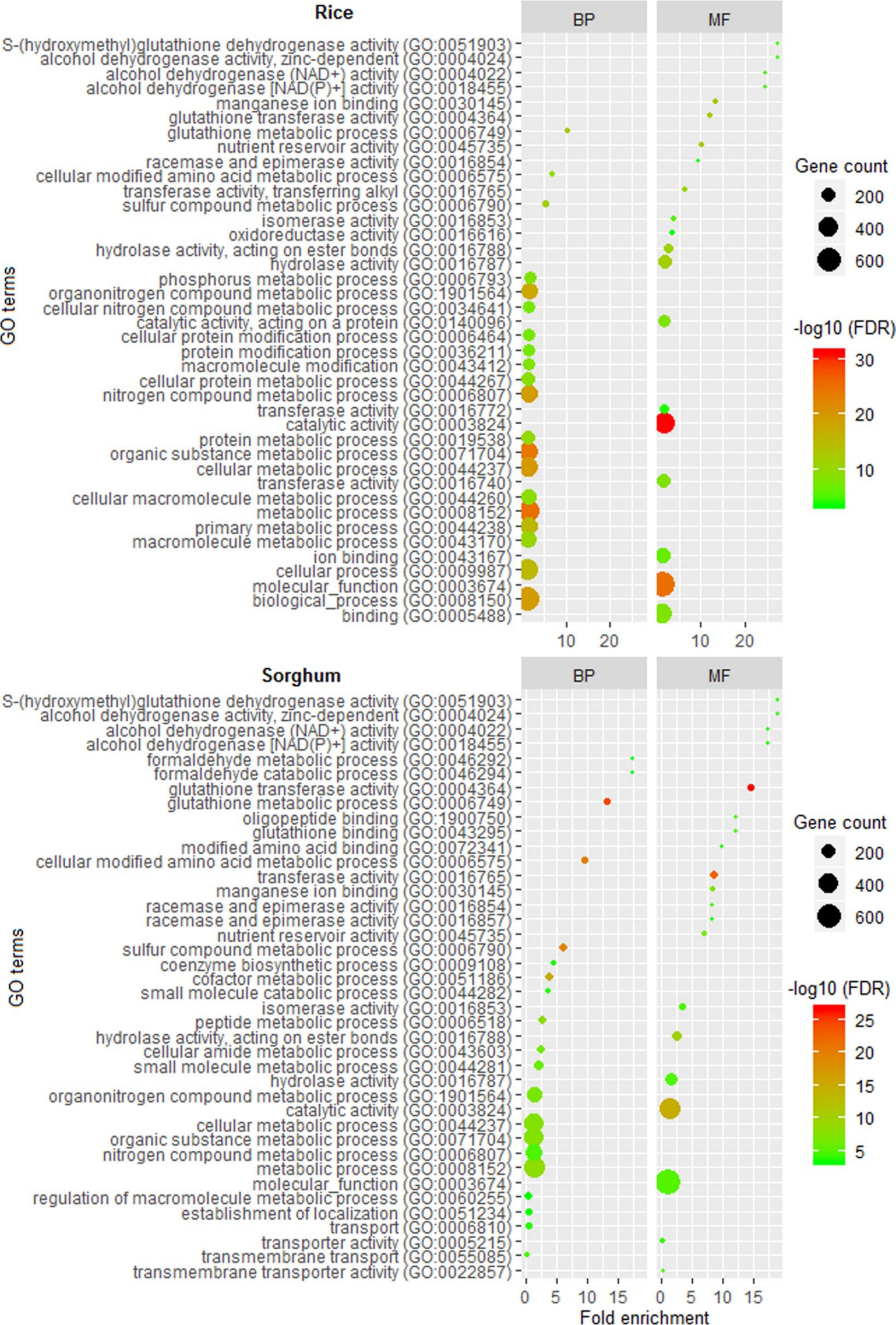
Gene Ontology terms analysis of the predicted circadian proteins for rice and sorghum

The developed computational model was further employed for proteome-wide identification of proteins associated with the CR (CR-proteins). We collected the proteome-wide sequence datasets of two crop species i.e., rice (proteme id: UP000059680) and sorghum (proteome id: UP000000768) from the proteome database (https://www.uniprot.org/proteomes/). There were four trained models in the background corresponding to Q1, Q2, Q3 and Q4. Based on the sequence length of the supplied test sequence, the trained model was first decided and subsequently the prediction was made. Out of 48,903 sequences of rice, only 1538 were predicted as CR-proteins with > 0.8 probability. Similarly, 1510 out of 41,298 sequences of sorghum were predicted as CR-proteins with > 0.8 probability. The probability threshold 0.8 was used to minimize the number of false positives. Functional analysis of the predicted 1538 rice sequences and 1510 sorghum sequences were also carried out with Gene Ontology (GO) terms. The GO annotation (biological process and molecular function) was performed using the PANTHER [76]. In rice, 1260 out of 1538 were mapped into biological processes (BP) and molecular functions (MF). In sorghum, 1140 out of 1510 were mapped into BP and MF. For BP in rice, biological_process (GO:0008150; 51.98%), cellular process (GO:0009987; 39.44%), metabolic process (GO:0008152; 38.57%), organic substance metabolic process (GO:0071704; 33.33%) and cellular metabolic process (GO:0044237; 31.19%) showed maximum number of hits (Fig. 4). With regard to MF in rice, the most represented GO terms were molecular_function (GO:0003674; 55.31%), catalytic activity (GO:0003824; 39.04%), binding (GO:0005488; 33.57%) and ion binding (GO:0043167; 20.79%) (Fig. 4). In sorghum, metabolic process (GO:0008152; 39.47%), organic substance metabolic process (GO:0071704; 33.15%), cellular metabolic process (GO:0044237; 32.11%) and nitrogen compound metabolic process (GO:0006807; 26.22%) were the most represented BP, whereas the molecular_function (GO:0003674; 57.36%), catalytic activity (GO:0003824; 40.78%) and hydrolase activity (GO:0016787; 14.12%) were the most represented MF (Fig. 4). The metabolic process showed significant enrichment in BP, whereas the catalytic, hydrolase and transferase activities were found significantly enriched for MF category in both rice and sorghum (Fig. 4).

### An R-package for users

Based on the proposed computational model, we developed an R-package “PredCRG” (https://cran.r-project.org/web/packages/PredCRG/index.html) for proteome-wide identification of proteins encoded by the circadian genes. There are three main functions in this package i.e., *PredCRG, PredCRG_Enc and PredCRG_training.* With the function *PredCRG,* users can predict the labels of the test protein sequences as circadian (CRG) or non-circadian (non-CRG) along with their probabilities. The function *PredCRG_Enc* can be used to encode the protein sequences based on the features of the PredCRG model. Most importantly, with the function *PredCRG_training*, users can develop their prediction models using four different kernel functions (Laplace, RBF, linear and polynomial) with their training datasets. The trained model can be subsequently used for the prediction of the test sequence of their interest. In summary, the developed R-package will be of great help for the researchers working in the field of identifying circadian genes via wet-lab experiments.

## Discussion

The distribution of common CR-related genes in plants is yet to be fully understood [63]. Identification of molecular components underlying the plant CR will certainly facilitate understanding the plant behavior in response to different environmental stimuli [77]. Circadian genes manipulation may help breeding crop cultivars with enhanced reproductive fitness [1, 33]. Circadian genes also reciprocate the defense signaling genes in plants [78]. Keeping in mind the roles of circadian genes, a computational model was developed in the present study to recognize the proteins encoded by the circadian genes.

We collected the experimentally validated circadian gene sequences of the plant species from the CGDB database (http://cgdb.biocuckoo.org/) and constructed the positive set. As far as non-circadian gene sequence is concerned, no database having such sequences is available. Thus, the protein sequences of the *Viridiplantae* clad collected from the UniProt database was used as the negative set. Further, we employed the CD-HIT algorithm to remove the redundant sequences from both the positive and negative sets. The CD-HIT algorithm sorts the input sequences from long to short, and processes them sequentially from the longest to the shortest. The first sequence is classified as the representative sequence of the first cluster. Then, each of the remaining sequences is compared to the representative sequences and is classified as redundant if it is found similar (with the given sequence identity cut-off) to the existing representative sequence. This process is repeated till all the sequences are classified as either redundant or representative. Finally, the non-redundant dataset (at the given threshold) is obtained by combining all the representative sequences. In this study, we applied a 40% sequence identity cut-off and obtained the dataset in which none of the sequences were > 40% identical to any other sequences.

The positive (39–4218 amino acids) and negative (43–5400 amino acids) datasets were found to be much diverse with regard to sequence length. As sequence length plays an important role in determining the physico-chemical properties of protein sequences, both positive and negative sets were partitioned into four homogeneous sub-datasets. As expected, improvements in accuracies were found with the homogeneous sub-datasets as compared to the heterogeneous full dataset. One of the probable reasons for this may be the generation of noisy observation vectors with the diverse sequence length. Amino acid composition and physico-chemical features of proteins determine their functions to a large extent [79–81]. Thus, the compositional and physico-chemical features were adopted for the generation of discriminative features.

The considered kernel functions are either expressed as the inner product of the feature vectors (polynomial, hyperbolic, linear and sigmoid) or the distance between the feature vectors (radial, Laplace and Bessel). Among the kernel functions, the Laplace kernel emerged as the best kernel followed by the linear and RBF for classification of circadian and non-circadian proteins. Though the Laplace kernel was found more appropriate in the present study, accuracy may vary with different positive and negative datasets.

**Fig. 5:**
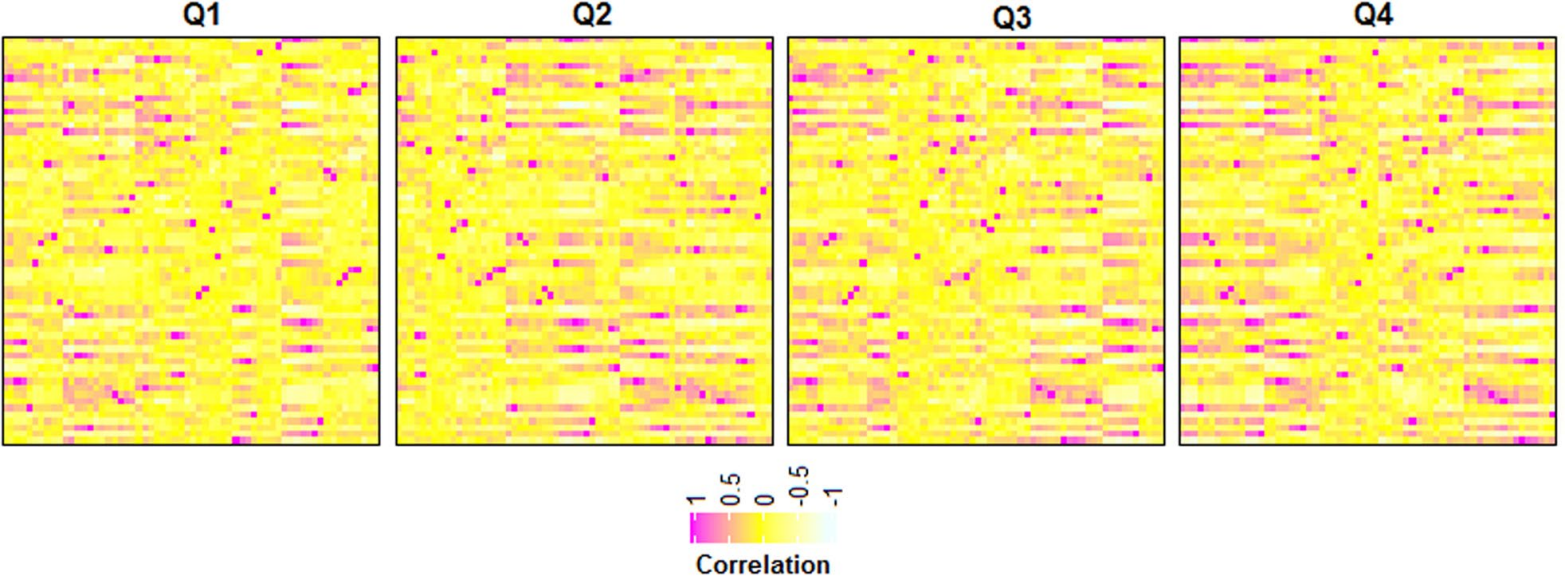
Heat maps showing the correlation among 62 numeric features in four sub-datasets. It can be seen that some of the features are highly correlated

While compared with other start-of-art machine learning methods such as RF, XGBoost, AdaBoost, Bagging, SVM was found to outperform them. We also noticed that the accuracy obtained with the LASSO was similar to that of SVM with the Laplace kernel. Although LASSO produces biased estimates, an advantage of LASSO is that it may yield higher accuracy by ignoring the redundant features. When we plotted the correlation matrix among the generated numeric features in the form of heat maps (Fig. 5), a higher degree of correlations was observed among certain features. The higher correlations among the features might have induced the redundancy in the feature set. So, one of the probable reasons for getting higher accuracy with the LASSO may be the use of only non-redundant features.

Motivated from the earlier studies [82, 83], the predicted label of the observation was utilized as additional feature. With the addition of such features, a little or no improvement in accuracy was found with the linear and Laplace kernels. On the other hand, improvement in accuracy was noticed with the RBF kernel. Improvement with the RBF and no improvement with the linear and Laplace kernels may be due to the non-linear relationship between the iteratively generated features (-1 s and 1 s only) and the response vector.

The developed computational model achieved ~ 63% classification accuracy, while assessed through fivefold cross-validation procedure. Similar accuracy was also obtained with the independent test dataset. Equivalent accuracy for five-fold cross-validation and independent test set implies that there was neither over-prediction nor under-prediction accuracy with the proposed model. We further performed proteome-wide identification of circadian proteins using proteome dataset of rice and sorghum, followed by the functional annotation of the predicted circadian proteins. For reproducibility of the work, we have developed the R-package “PredCRG”. We anticipate that this package would not only be helpful for the users to predict their test sequences, but also to build their prediction model using their training dataset.

## Conclusions

This study presents a novel computational approach for the recognition of proteins encoded by the circadian genes. The prediction accuracy is not very high. However, this is the first computational approach for predicting the circadian genes (proteins) with the sequence dataset. So, we believe that further improvement can be made by including more discriminatory feature sets. The developed approach is expected to supplement the existing models that are based on gene expression data. The R-package “PredCRG” is believed to be of great help to the scientific community for proteome-wide identification of circadian genes. Our future endeavor would be to develop a more accurate model by using the sequence dataset.

## Supplementary Information

**Additional file 1: Table S1.** Summary of the numeric feature sets and the R-packages used to generate these features.

**Additional file 2: Table S2.** Default parametric values for different kernel functions and R-packages used for execution of support vector machine with different kernel functions.

**Additional file 3: Table S3.** R-packages, functions and the parametric values used for execution of different machine learning algorithms.

## Data Availability

All the datasets used in this study are available at https://github.com/meher861982/PredCRG_dataset.

## Abbreviations

CR: Circadian rhythm
CC: Circadian clock
SVM: Support vector machine
AdaBoost: Adaptive boosting
XGBoost: Extreme gradient boosting
LASSO: Least absolute shrinkage and selection operator
GO: Gene ontology
AAC: Amino acid composition
FASGAI: Factor analysis scales of generalized amino acid information
RBF: Radial basis function
ROC: Receiver operating characteristic
PR: Precision-recall
auROC: Area under ROC curve
auPRC: Area under PR curve
MCDM: Multiple criteria decision making
TOPSIS: Technique of order preference similarity to the ideal solution
NCBI: National center for biotechnology information
RF: Random forest
MF: Molecular function
BP: Biological process
TPR: True positive rate
TNR: True negative rate
PPV: Predictive positive value
