# Supplementary material for: PredCRG: A computational method for recognition of plant circadian genes by employing support vector machine with Laplace kernel
Source: Plant Methods. 2021 Apr 26;17:46. doi: 10.1186/s13007-021-00744-3 (PMC8074503; doi:10.1186/s13007-021-00744-3)
Supplement: Supplementary file 1 — Additional file 1: Table S1. Summary of the numeric feature sets and the R-packages used to generate these features. [file 13007_2021_744_MOESM1_ESM.docx]

**Table S1**. Summary of the numeric feature sets and the R-packages used to generate these features.

| **Feature type** | **Description** | **#Features** | **R-package used** |
| --- | --- | --- | --- |
| Amino acid composition (AAC) | This constitutes the frequencies of 20 amino acids present in protein sequence. | 20 | *protr* |
| FASGAI  (Factor Analysis Scales of Generalized Amino Acid Information) | It is a set of numeric features generated for each protein sequence, which reflects the hydrophobicity, alpha and turn propensities, bulky properties, compositional characteristics, local flexibility and electronic properties. | 6 | *Peptides* |
| Cruciani properties | It is the scaled principal component scores that summarize a set of descriptors computed based on the interaction of each amino acid residue with different chemical groups. | 3 | *Peptides* |
| ProtFP | It comprises the first 8 principal components of 58 AAindex properties of 20 amino acids. | 8 | *protr* |
| Transitional features | It includes the frequency of one type of amino acid followed by other types of amino acid. In this study, 8 types of residues were employed i.e., polar, neutral, hydrophobic, strand, helix, coil, exposed and buried. The first three types of residues are with regard to hydrophobicity, the next three types are with respect to secondary structure and the last two are corresponding to solvent accessibility. | 21 | *protr* |
| Physico-chemical properties | Hydrophobicity, Instability index, Molecular weight and iso-electric point | 4 | *Peptides* |
