# Supplementary material for: PredCRG: A computational method for recognition of plant circadian genes by employing support vector machine with Laplace kernel
Source: Plant Methods. 2021 Apr 26;17:46. doi: 10.1186/s13007-021-00744-3 (PMC8074503; doi:10.1186/s13007-021-00744-3)
Supplement: Supplementary file 2 — Additional file 2: Table S2. Default parametric values for different kernel functions and R-packages used for execution of support vector machine with different kernel functions. [file 13007_2021_744_MOESM2_ESM.docx]

**Table S2**. Default parametric values for different kernel functions and R-packages used for execution of support vector machine with different kernel functions.

| **Kernel function** | **Parameters** | **R-package** |
| --- | --- | --- |
| Radial | $\gamma=\frac{1}{\#Features}$ | *e1071* |
| Polynomial | $\gamma=\frac{1}{\#Features}$  $r=0$  $d=3$ | *e1071* |
| Linear | $C=1$ | *e1071* |
| Hyperbolic | $\gamma=1$  $r=1$ | *kernlab* |
| Laplace | $\gamma=1$ | *kernlab* |
| Bessel | $\gamma=1$  $Order=1$  $d=1$ | *kernlab* |
| Sigmoid | $r=1$  $d=1$ | *kernlab* |

$d:$ degree; $r:$ offset; *C*: cost
