# Supplementary material for: PredCRG: A computational method for recognition of plant circadian genes by employing support vector machine with Laplace kernel
Source: Plant Methods. 2021 Apr 26;17:46. doi: 10.1186/s13007-021-00744-3 (PMC8074503; doi:10.1186/s13007-021-00744-3)
Supplement: Supplementary file 3 — Additional file 3: Table S3. R-packages, functions and the parametric values used for execution of different machine learning algorithms. [file 13007_2021_744_MOESM3_ESM.docx]

**Table S3.** R-packages, functions and the parametric values used for execution of different machine learning algorithms.

| **Machine learning algorithm** | **R-package** | **function** | **Parameters** |
| --- | --- | --- | --- |
| Random Forest | randomForest | *randomForest* | ntree=500, mtry=$\sqrt{\#Feature}$ |
| Bagging | ipred | *bagging* | *nbagg=25* |
| AdaBoost | adabag | *boosting* | *mfinal = 100,*  *coeflearn = 'Breiman'* |
| XGBoost | xgboost | *xgboost* | *max_depth = 2, eta = 1, nthread = 2, nrounds = 2* |
| LASSO | glmnet | *glmnet* | *family=“binomial”, alpha=1, nlambda=100, lambad=lambda.min* |
